# Supplementary material for: An efficient and scalable pipeline for epitope tagging in mammalian stem cells using Cas9 ribonucleoprotein
Source: eLife. 2018 Apr 11;7:e35069. doi: 10.7554/eLife.35069 (PMC5947990; doi:10.7554/eLife.35069)
Supplement: Transparent reporting form [file elife-35069-transrepform.docx]

***eLife’s* transparent reporting form**

We encourage authors to provide detailed information *within their submission* to facilitate the interpretation and replication of experiments. Authors can upload supporting documentation to indicate the use of appropriate reporting guidelines for health-related research (see [EQUATOR Network](http://www.equator-network.org/%20)), life science research (see the [BioSharing Information Resource](https://biosharing.org/)), or the [ARRIVE guidelines](http://www.plosbiology.org/article/info:doi/10.1371/journal.pbio.1000412) for reporting work involving animal research. Where applicable, authors should refer to any relevant reporting standards documents in this form.

If you have any questions, please consult our Journal Policies and/or contact us: [editorial@elifesciences.org](mailto:editorial@elifesciences.org).

**Sample-size estimation**

- You should state whether an appropriate sample size was computed when the study was being designed
- You should state the statistical method of sample size computation and any required assumptions
- If no explicit power analysis was used, you should describe how you decided what sample (replicate) size (number) to use

Please outline where this information can be found within the submission (e.g., sections or figure legends), or explain why this information doesn’t apply to your submission:

We did not use power analysis in our study because we believe that the sample size parameter would not have any impact on our findings. We wanted to optimize a Cas9 RNP-based epitope knock-in method in neural and glioma stem cells. This was performed on various stem cell lines derived from mouse and human samples. The efficacy and efficiency of knock-in assay does not depend on the sample size, therefore, we did not use any power analysis.

**Replicates**

- You should report how often each experiment was performed
- You should include a definition of biological versus technical replication
- The data obtained should be provided and sufficient information should be provided to indicate the number of independent biological and/or technical replicates
- If you encountered any outliers, you should describe how these were handled
- Criteria for exclusion/inclusion of data should be clearly stated
- High-throughput sequence data should be uploaded before submission, with a private link for reviewers provided (these are available from both GEO and ArrayExpress)

Please outline where this information can be found within the submission (e.g., sections or figure legends), or explain why this information doesn’t apply to your submission:

In the figure legends, we have mentioned how many times each experiment was performed. For the comparison of knock-in efficiency achieved using in vitro transcribed RNA versus synthetic RNA in figure 1, a minimum of two independent experiments were carried out for each gene. For rest of the experiments, wherein we wanted to assay large set of crRNAs against many target genes, we performed a single experiment for each crRNA. However, for each set of these experiments, a positive control crRNA against Sox2 gene knock-in was always included to assess experiment to experiment variability.

**Statistical reporting**

- Statistical analysis methods should be described and justified
- Raw data should be presented in figures whenever informative to do so (typically when N per group is less than 10)
- For each experiment, you should identify the statistical tests used, exact values of N, definitions of center, methods of multiple test correction, and dispersion and precision measures (e.g., mean, median, SD, SEM, confidence intervals; and, for the major substantive results, a measure of effect size (e.g., Pearson's r, Cohen's d)
- Report exact p-values wherever possible alongside the summary statistics and 95% confidence intervals. These should be reported for all key questions and not only when the p-value is less than 0.05.

Please outline where this information can be found within the submission (e.g., sections or figure legends), or explain why this information doesn’t apply to your submission:

We used student’s unpaired t test to compare knock-in efficiency achieved with IVT gRNA versus synthetic cr/tracrRNAs in the figure 1. The P values are indicated in the main figure, error bars representing standard deviation between two or three independent experiments are also shown.

(For large datasets, or papers with a very large number of statistical tests, you may upload a single table file with tests, Ns, etc., with reference to sections in the manuscript.)

**Group allocation**

- Indicate how samples were allocated into experimental groups (in the case of clinical studies, please specify allocation to treatment method); if randomization was used, please also state if restricted randomization was applied
- Indicate if masking was used during group allocation, data collection and/or data analysis

Please outline where this information can be found within the submission (e.g., sections or figure legends), or explain why this information doesn’t apply to your submission:

This does not apply to our submission because our experiments did not involve experimental groups. We tested efficiency of epitope knock-in at the C-terminus of many genes in different stem cell types, no experimental groups were used for these assays.

**Additional data files (“source data”)**

- We encourage you to upload relevant additional data files, such as numerical data that are represented as a graph in a figure, or as a summary table
- Where provided, these should be in the most useful format, and they can be uploaded as “Source data” files linked to a main figure or table
- Include model definition files including the full list of parameters used
- Include code used for data analysis (e.g., R, MatLab)
- Avoid stating that data files are “available upon request”

Please indicate the figures or tables for which source data files have been provided:

We have submitted four supplementary excel sheets.

Supplementary Table 1: Contains the sequences of crRNAs and ssODN donor DNA templates used in this study.

Supplementary Table 2: Contains the sequences of PCR primers used in this study.

Supplementary Table 3: Contains the information about the knock-in efficiency achieved with the 96-well format transfections. Name of the genes and corresponding knock-in efficiencies are displayed in column format.

Supplementary Table 4: Contains the list of protein interaction partners of Olig2 as identified using RIME and ChIP-SICAP methods.
